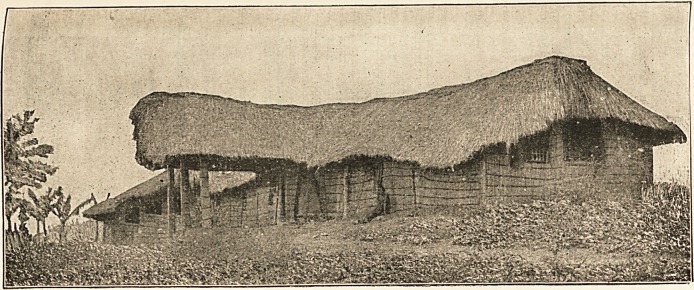# Scraps

**Published:** 1898-12

**Authors:** 


### SCRAPS

PICKED UP BY THE ASSISTANT-EDITOR.

**Clinical Records** (25).—A magistrate of Edinburgh, contemporary with "Lang Sandy Wood," the eminent physician, planned how to get from the latter a prescription without a fee. Taking advantage of a custom of the time, he invited Sandy to take his meridian with him in a "change house" near the Cross. Over the wine he gave a long account of his ailments, to which Wood listened in grim silence. At last he put the direct question: "Doctor, what do you think I should tak'?" "Tak'!" exclaimed Sandy, "why, if ye're as ill as ye say, I think ye should tak' medical advice."—*Medical Age.*

**Medical Philology** (XXVIII.)—The *Promptorium* gives "Dalke. *Vallis*." *Vallis* is also given as the Latin equivalent of the English "dale, or vale." Mr. Way in a note says: "Delk, according to Forby [*The Vocabulary of East Anglia*, 1830—38], signifies in Norfolk a small cavity either in the soil, or the flesh of the body. In this last sense the gloss on Gautier de Bibelesworth [1325] interprets the expression '*au cool troueret la fosset*, a dalke in þe nekke.' Arund. MS. 220, f. 297, b."

The etymology of the word is unknown. *The New English Dictionary* thinks it may be a diminutive of dale or dell, and cites the Frisian *dölke*, a dimple, as a diminutive of *döle*, a hollow.

There was another sense in which the word "dalke" was used. It occurs in the *Catholicon* as meaning a pin or brooch. Mr. Herrtage points out that in this case its origin arose in *dalkr*, old Icelandic for a thorn.

**Medical Knowledge.**—Dr. W. D. Hamaker, a member of the Pennsylvania State Board of Examiners, records some answers given by doctors in the examination for the State license to practise. The following are some of the most striking: "Hydrogen gass is degenerated from the urea." "Cantharides is derived from the root of the plant." "Pix liquida is from the Pinus Somniferous group." "Cantharides is derived from the destructive distillation of the Spanish fly." "The malar bone articulates with the occipital bone." "Picrotoxin is an alkaloid of senna and rhubarb." "Spartein and eserine are alkaloids of somnis papverum." One said that "Spartein was derived from Sparta," and another said it "was derived from Spartus." Another said that "vinegar was an antidote for mineral acid poisons;" and another stated that "an infusion of Spanish flies was one of the official preparations." Another gave us the information that "the uriniferous tubules secrete the seminal fluid." Another said that "belladonna locks up all of the secretions except the urine and feces." Another stated, without additional matter of any kind, that "the differential diagnosis between epilepsy and hysteria is that in epilepsy they fall on the stove and burn themselves, and in hysteria they don't." Turning to obstetrics we found one man who, in rigid os, "would decapitate or perform craniotomy, or would put on the forceps and deliver at once." Another, in performing version, "would put his finger in the child's mouth and bring the chin under the os pubis and hold his hand over the mouth to prevent the liquor amnia from choking it." Another stated that "the endocardium is a mucous membrane which weighs 2 oz., and is separated from the pleura by the pericardium." Another stated that "the function of the optic nerve is to contract the pupil and move the eyeball." A new diagnostic symptom was offered by another man, whose paper stated that "in cerebral hemorrhage the patient may vomit the cerebro-spinal fluid!" And yet all these men have diplomas!—*Pennsylvania Medical Journal*, quoted in *St. Louis Medical and Surgical Journal.*

**"The Physician, his Personnel, and How it Affects his Success."**—In an article with this title, the whole of which is worth reading, Dr. T. J. Hillis says "the well-disciplined physician will not worry too much if things do not go to his liking in the sick-room, or take it to heart if he loses a patient suddenly and unexpectedly. If he does, the sooner for his peace of mind and health he enters another pursuit the better for him. The constant fretting will wear his mind and consume his body, and while yet young he shall be gathered to his fathers and join the majority beyond.

"The wise physician will forget the pains and tribulations of the sick-room after the door is closed behind him. In that room let him think and use his skill and judgment well, give his instructions in an easy yet emphatic manner, not recapitulate except on special occasions. Often for this cause good doctors have been accounted bores, and patients have disappeared from their lists without their knowing why.

"The physician must not be too sympathetic in the sick-room, or carried away by the tears and sad faces he sees around him. He must be like the sturdy oak in the forest—bend to the blast, while not being affected by it. He must always keep himself well in hand, never lose his temper or presence of mind; if he is master of himself, he will seldom have difficulty in being master of the sick-room. He must be ever conscious of the fact that the family did not send for him for the purpose of sympathizing with them; their friends and spiritual adviser are abundantly able to do this, and more too, for it will be found that the former, and unfortunately often the latter, indulge in criticisms not overfavorable to the physician. His method of treatment will be measured by his degree of success, and, if failure perches on his banner, a cute and knowing friend in the background comes to the front to whisper into the ear of the distressed relative: 'I told you so; I said all along he did not understand the case.'

"The nature and character of the disease play no part with these people. It is always a question of the degree of ability of the physician. . . . When they are convinced or imagine some one else can do better, they throw him down like a dishrag."—*Medical Record.*

**As Others See Us.**—In welcoming the American Medical Association at its Annual Meeting in Denver, the Honourable Alva Adams, Governor of Colorado, said: ". . . In ancient India only the most noble of Brahmins was allowed to practise medicine. As we read the splendid faces of those who compose this Congress we can half believe that the influence of the ancient law has not been lost. Royal or not, I have always admired the regal way in which doctors ignore their own advice and rule. As I came upon the platform, your secretary said to me, 'Treat this audience kindly, but do not take their medicine.' We know that preachers sometimes obey their own injunctions, but doctors never. Go into their homes, you run against no rules of diet, meals, beverage, sleep; all is liberty and happiness. Perhaps they read with a clearer vision that brief but suggestive Biblical obituary, 'Asa trusted the physicians and he slept with his fathers.' . . .

"If kindness to misfortune, sacrifice for the poor, be a pass to paradise, I would rather take the chance of many a poor Dr. McClure than stand in the shoes of some of our proud millionaires. But with all of your ability, with all of your generosity, which is equaled only by your skill, I find that the doctors are a modest set of people. Doctors are very tolerant, indulgent and generous, unless called to consult with some one of another school. With my experience in legislative matters, and in the discussion of legislative bills which had to do with the regulation of practice and the recognition of different schools of medicine, it has given birth to a suspicion that the first fundamental principle of each school is that the others had no business to practise; that they ought to be prohibited or go to jail. Personally, my condition has much to do with my faith in schools and systems. For instance, if I feel lonesome and forsaken; if the newspapers, and politicians, and the disappointed, turn their pens, tongues and scalping knives upon me, just because I was not so wise as they would be in the conduct of my office and in making appointments, then I feel the need of the soothing, sympathetic treatment of the Christian scientist or faith cure. Again, if I am in what you may call the loafing, novel reading degree of invalidism, when it does not hurt very bad, I call in my homeopathic friends. Their remedies seem as pleasant as their gentle touch and manners. Their dissertations upon the power of atoms are as fascinating and convicting as a chapter from Tyndall or Hugh Miller. But, my friends, so strong is the power of ancient usage, so strong is the memory of youth, and the influence of early training, that when I have an ache, when I feel that there is some chance of my account being called to a close, then I send for the old regular calomel doctor, and I want him quick." —*Journal of the American Medical Association.*

**A Grass Hospital.**—This picture of Mengo Hospital in Uganda needs a few words of explanation as to the location of the various wards and rooms. The long building with the porch is the men's ward. Beyond it, with three windows, is the operating theatre, which is a room opening out of the women's wards. Beyond it again is the spare ward, which is used for either male or female patients when the regular wards are all full. The operating theatre is lighted by the three windows, and the table is just inside, under the windows, with room to pass between it and the wall. Mr. R. A. Leakey, who is a member of the Uganda Mission, writes thus: "I am sure you will see the need there is of a new hospital of brick or something less germ-retaining than a grass building. Now that the whole Nile Valley is under the British, and we have a good hospital at one end (Cairo), and money for a good one half-way (Khartoum), surely the other end must not be neglected."—*Mercy and Truth.*

Who will help? Money should be sent to Dr. H. LANKESTER, C.M.S., Salisbury Square, London, E.C.

**Molière and the Doctors.**—A friend of mine has kindly written for me the following, which I am sure will interest my readers:

Molière's fierce antipathy to the Leeches and Barber-surgeons of his time is well known. Of the six plays[1] in which he lashes them, the best known are *Le Médecin malgré lui* and *Le Malade imaginaire*. In the former, the satire is indeed oblique; for Sganarelle is after all but an ignorant faggot-maker. Yet the extreme ease with which he personates a doctor, and his delicious mimicry of the crass ignorance and the limitless assurance of the faculty, make every hit palpable; *e.g.*, Jacqueline: "Je me porte le mieux du monde." Sga.: "Tant pis, tant pis. Cette grande santé est à craindre . . . il faut se faire aussi saigner pour la maladie à venir." (ii. 5.) Again, Géronte: "Il me semble . . . que le cœur est du côté gauche, et le foie du côté droit." Sga.: "Oui, cela était autrefois ainsi; mais nous avons changé tout cela." (ii. 4.)

In *Le Malade imaginaire* all reserve is dropped: patient and doctor alike are scourged with an energy truly Aristophanic. Argan, a robust hypochondriac, discovers by his apothecary's bills that he has taken but eight prescriptions this month, as against twelve last month, and exclaims, "Je ne m'étonne pas si je ne me porte pas si bien ce mois-ci que l'autre. Je le dirai à M. Purgon, afin qu'il mette ordre à cela." (i. 1.) He inquires of the doctor how many grains of salt he is to put into an egg, and is advised to use some even number, "comme dans les médicaments par les nombres impairs." (ii. 9.) He is afraid so much as to lift his cap at one doctor's entrance; for says he, "M. Purgon m'a défendu de découvrir ma tête. Vous êtes du métier; vous savez les conséquences." (ii. 6.) When ordered to take twelve turns in his room, he is in despair because he forgot to ask whether he was to pace the room up and down, or athwart!

Dr. Diafoirus's principle is "On n'est obligé qu'à traiter les gens dans les formes: c'est à eux à guérir s'ils peuvent." He is so conservative as to speak scorn of "les prétendues découvertes de notre siècle, touchant la circulation du sang, et autres opinions du même farine." (ii. 6.) Béralde suggests how economical a thing it were, if Argan got himself admitted a practitioner. Arg.: "Mais il faut savoir bien parler latin." Bér.: "En recevant la robe et le bonnet de médecin vous apprendrez tout cela." Arg.: "Quoi! l'on sait discourir sur les maladies quand on a cet habit-là?" Bér.: "Oui. L'on n'a qu'à parler avec une robe et un bonnet, tout galimatias devient savant, et toute sottise devient raison." (iii. 22.) But how to describe the final "Intermède"? Enter 8 syringists (porte-seringues), 6 apothecaries, and 22 doctors. They finally settle down to examine in atrocious Latin a candidate for the M.B. Asked for his treatment of dropsy, the latter replies, "Clysterium donare, Postea seignare, Ensuita purgare": this extorts from the examiners, "Dignus, dignus est intrare In nostro docto corpore." For phthisis, the same treatment, the same approval. Lastly, this supposititious case:

"Habet grandam fievram cum redoublamentis
Grandam dolorem capitis,
Et grandum malum au côté,
Cum granda difficultate
Et pena à respirare."

Same treatment; but if that fail, "Reseignare, repurgare et reclysterisare." Whereupon the lucky student obtains with honour

"Virtutem et puissanciam
Medicandi,
Purgandi,
Seignandi,
Percandi,
Taillandi,
Coupandi,
Et occidendi
Impune per totam terram."

Molière, while acting in this burlesque scene in this his latest play, was struck with convulsions, and died within a few hours; sealing by his death his undying hatred of the Faculty.

1 *Le Médecin volant; Le Docteur amoureux; L'Amour médecin; Le Médecin malgré lui; M. de Pourceaugnac; Le Malade imaginaire.*

## Figures and Tables

**Figure f1:**